# RETRACTION: Depletion of lncRNA MEG3 Ameliorates Imatinib‐Induced Injury of Cardiomyocytes via Regulating miR‐129‐5p/HMGB1 Axis

**DOI:** 10.1155/ancp/9761979

**Published:** 2026-06-12

**Authors:** Analytical Cellular Pathology

RETRACTION: P. Tang, J. Zhou, H. Liu, S. Mei, K. Wang, and H. Ming, “Depletion of lncRNA MEG3 Ameliorates Imatinib‐Induced Injury of Cardiomyocytes via Regulating miR‐129‐5p/HMGB1 Axis,” *Analytical Cellular Pathology*, 2023, 1108280, https://doi.org/10.1155/2023/1108280.

The above article, published online on 17 November 2023 in Wiley Online Library (wileyonlinelibrary.com), has been retracted by agreement between the Journal Editor‐in‐Chief, Dimitrios Karamichos; and John Wiley & Sons Ltd. The retraction has been agreed due to concerns identified with Figure 1e. Specifically, when resized and flipped horizontally, the Cleaved Caspase 3 bands in Figure 1e appear highly similar to the mTOR/MCF‐7 bands shown in Figure 5 of an article by different authors [1].

Moveover, concerns were reported on PubPeer [2] regarding the apoptotic measurements in Figures 1 and 2.

The authors did not provide a response to the concerns, and after assessment by the Editorial Board the results and conclusions cannot be considered reliable. The article is therefore retracted.

The authors did not respond to the retraction.
